# AiRobSim: Simulating a Multisensor Aerial Robot for Urban Search and Rescue Operation and Training

**DOI:** 10.3390/s20185223

**Published:** 2020-09-13

**Authors:** Junjie Chen, Shuai Li, Donghai Liu, Xueping Li

**Affiliations:** 1 Department of Civil and Environmental Engineering, The University of Tennessee, Knoxville, TN 37902, USA; chenjj@tju.edu.cn; 2 State Key Laboratory of Hydraulic Engineering Simulation and Safety, Tianjin University, Tianjin 300350, China; liudh@tju.edu.cn; 3 Department of Industrial and Systems Engineering, The University of Tennessee, Knoxville, TN 37902, USA; Xueping.Li@utk.edu

**Keywords:** situational awareness, search and rescue, robot simulation, ground-penetrating radar, unmanned aerial vehicle, training

## Abstract

Unmanned aerial vehicles (UAVs), equipped with a variety of sensors, are being used to provide actionable information to augment first responders’ situational awareness in disaster areas for urban search and rescue (SaR) operations. However, existing aerial robots are unable to sense the occluded spaces in collapsed structures, and voids buried in disaster rubble that may contain victims. In this study, we developed a framework, AiRobSim, to simulate an aerial robot to acquire both aboveground and underground information for post-disaster SaR. The integration of UAV, ground-penetrating radar (GPR), and other sensors, such as global navigation satellite system (GNSS), inertial measurement unit (IMU), and cameras, enables the aerial robot to provide a holistic view of the complex urban disaster areas. The robot-collected data can help locate critical spaces under the rubble to save trapped victims. The simulation framework can serve as a virtual training platform for novice users to control and operate the robot before actual deployment. Data streams provided by the platform, which include maneuver commands, robot states and environmental information, have potential to facilitate the understanding of the decision-making process in urban SaR and the training of future intelligent SaR robots.

## 1. Introduction

After natural and man-made disasters, rapid search and rescue (SaR) operations need to be conducted to save surviving victims [1,2]. However, in urban areas, the unstructured, occluded and complex disaster site poses challenges and risks to first responders during SaR operations. To enable efficient, effective and safe urban SaR operations, robots are being developed to assist first responders by offering unprecedented situational awareness about disaster areas. For example, unmanned aerial vehicles (UAVs) and unmanned ground vehicles (UGVs) have been used to generate three-dimensional (3D) maps of disaster areas [3,4], search for survivors [5,6], and evaluate property damages [7,8]. Surviving victims are often entrapped in voids that are buried in heterogeneous rubble. The UAVs and UGVs, equipped with vision-based sensors such as cameras and light-detection and ranging (LiDAR), can obtain information about the surface of disaster areas, but are unable to sense the occluded spaces under rubble to provide first responders with actionable information. Snake robots [9] can enter collapsed structures to search for survivors. However, their search efficiency is limited due to the low moving speed in rubble-strewn structures. There remains, therefore, a critical need to develop a new robot that can collect both the above-rubble and below-rubble information in a reliable and timely fashion to expedite SaR operations. In the absence of such a solution, the occluded, complex, and hazardous environments will continue to hinder SaR operations, resulting in injuries and fatalities of both victims and first responders [10].

Ground-penetrating radar (GPR) is an effective technique for subsurface detection based on electromagnetic signal processing. The feasibility of using GPR for SaR operations has been demonstrated in a few pilot studies [11,12,13]. Integrating GPR with robotic platforms such as UAV can take advantage of the high mobility and flexibility of drones while utilizing the capability of GPR in underground sensing. Due to its merits, such a UAV-GPR hybrid setup has been investigated. Previous research mainly focused on the hybrid use of UAV and GPR in detecting avalanche victims [13,14,15]. Compared with SaR in snowfield, urban SaR operations are more challenging because urban disaster sites are usually highly occluded and obscured. The challenge calls for research efforts to investigate the applicability of airborne GPR in assisting urban SaR.

In the context of urban search and rescue, simulation is an important tool for robot design and operator training. The rationale is two-fold. First, using simulation to test and evaluate the performance of a newly designed robot in virtual disaster areas is cost-effective, and will provide valuable insights for deploying it in real-world scenarios. Second, first responders need adequate training before they can proficiently and effectively work with the newly designed robot in real disaster areas. With various authentic disaster scenarios, the interaction with the robot and virtual disaster areas in the high-fidelity simulation platform will allow users to gain skills in operating the robot and in interpreting the robot-collected data to conduct SaR operations.

Motivated by the above, this study devised an aerial SaR robot by the integration of UAV and GPR to obtain both the above-rubble and below-rubble information in urban environments, and developed a simulation framework, AiRobSim, for robot testing and user training in various disaster scenarios. The contribution of this study is two-fold. First, the hybrid use of UAV and GPR is adopted in urban search and rescue to provide first responders with holistic situation awareness of the occluded, and complex urban disaster areas, with which critical void locations that may contain surviving victims can be pinpointed. The setup of the robot can allow it to both navigate over the complex urban disaster site easily and map the underground condition rapidly, thus helping expedite the urban SaR operations. Second, various disaster scenarios were built in a virtual environment, and different collapse patterns such as pancake, lean-to, V-shape, and A-frame were considered to reflect the real-world scenarios and to maintain high fidelity of the simulations. In addition, an interface was developed for interactive human–robot communication and control, which enables novice users to quickly acquaint themselves with knowledge and skills in operating the robot.

## 2. Background Review

### 2.1. Search and Rescue Robots

Aerial and ground robots have been used in post-disaster search and rescue. Unmanned aerial vehicles (UAVs) are effective platforms for data acquisition in disaster areas. Recchiuto and Sgorbissa [8] reviewed how UAVs can be used in post-disaster assessment, and discussed various topics including aerial platforms, multirobot software architectures, onboard sensors, and simultaneous localization and mapping (SLAM) methods. Fixed-wing UAVs are normally used at the initial survey stage due to the merits of fast moving speed and large sensing coverage [7,16,17], while mini helicopters and multi-rotors are being used for near-ground search and indoor application [3,18,19] because they are easy to operate and can hover at a fixed point. Rudol and Doherty [6] exploited color and thermal image data collected by UAVs to detect human bodies. Their method can visually geolocate the positions of the detected objects or human bodies on an aerial map. Arnold et al. [20] used a swarm of autonomous flying robots equipped with cameras and heartbeat-locator sensors to gain situational awareness after a natural disaster. Kochersberger et al. [21] developed a remote sensing system for urban disaster assessment and relief by incorporating a stereovision system, an onboard spectrometer, and a tethered robot into an autonomous helicopter platform.

Multiscale ground robots have also been deployed in disaster areas. Large ground robots usually have a large payload, allowing them to carry multiple sensors and relief supplies. Small ground robots can enter collapsed structures, which are not accessible to traditional aerial robots. Unmanned ground vehicles (UGVs) have been used in SaR operations for many years. For example, UGVs were deployed to search for survivors after the 9/11 attack [5], and used to assess the level of radiation inside damaged nuclear power plants in the 2011 Japan earthquake [22,23]. To improve UGVs’ locomotion performance on rough terrain, many autonomous control algorithms have been proposed for obstacle avoidance and self-correction [4,24]. However, these robots fall short of operating in narrow and cluttered spaces, which are common in urban disaster areas. To explore the narrow spaces under rubble, Ito et al. [25,26] developed a snake-like robot with high maneuverability by applying passive mechanisms. Hawkes et al. [9] developed a soft pneumatic robot by imitating the pattern of movement found in developing neurons and fungal hyphae. The robot has the capability of moving through constrained and cluttered spaces, such as the rubble-strewn environment in disaster areas.

### 2.2. Ground-Penetrating Radar in Search and Rescue

As an effective tool for subsurface sensing, ground-penetrating radar (GPR) has been applied in many different areas, such as archaeology and geological prospecting. Like these areas, research on the application of GPR in SaR has also been ongoing for years. According to the sensing rationales they rely on, current research efforts can be divided into two aspects. One line of work intends to use the electromagnetic wave emitted by GPR to capture the respiration movements or physical motion of trapped victims, thus enabling the detection of buried survivors. Li et al. [27] and Yarovoy et al. [28] developed techniques to process ultra-wideband (UWB) radar signals for the identification of a human’s vital signs (e.g., breathing and heartbeat) in post-disaster environments. Hamp et al. [29] provided a continuous wave GPR that is capable of detecting the respiration of survivors trapped in collapsed buildings. Due to the limited penetration depth of UWB, research based on respiration detection is difficult to fulfill its potential in practical SaR scenarios, where the depth of trapped victims can range from 1 to 10 m.

The other line of research tries to pinpoint locations of buried victims via the reflection strength of electromagnetic signals by different materials. It was found that interface between two materials with significantly different dielectric constant tends to reflect more electromagnetic signals, resulting in more dramatic features in the corresponding GPR scans. Based on this observation, research attempts have been made to identify avalanche victims from collected GPR profiles, e.g., Heilig [11], Instanes et al. [12], and Fruehauf et al. [13]. Victim detection in urban scenarios can follow the similar rationale, because void spaces filled with air under the rubble have a significantly different dielectric constant than the collapsed concrete materials. However, the complex, heterogeneous urban post-disaster environments pose greater challenges, compared with the relatively simple and homogeneous snow in avalanche scenarios. Hence, there is a need to investigate whether GPR is applicable in urban SaR operations for effective void recognition.

To improve the efficiency of GPR scanning, the integration of GPR and UAV is a research direction gaining more and more attention. Machguth et al. [30] used helicopter-borne GPR for the investigation of snow accumulation, while Arcone and Delaney [31] employed the GPR-UAV hybrid setup to identify hidden crevasses in Antarctica. In SaR operations, such integration has mainly been reported in the searching of avalanche victims [13,14,15]. Since a damaged urban environment is much more occluded and complex than the snowfield, it remains unclear whether the GPR-UAV hybrid setup will be considered feasible in urban SaR.

### 2.3. Robot Simulation in Virtual Environment

Simulation has recently gained momentum in the robotics research community. One line of work focuses on using simulations to test robot operation algorithms or tune control parameters. Shamshiri et al. [32] conducted a review on open-source simulation software, and pointed out that simulation plays an important role in robot debugging and performance optimization. Couceiro et al. [33] tested their proposed algorithms for multi-robot SaR tasks in a simulated environment. Hayat et al. [34] proposed a multi-objective path planning algorithm for SaR, and tested it with a UAV swarm in simulation. Similar studies also include References [35,36]. Ding et al. [37] developed a virtual prototype of a hydraulic exoskeleton robot in simulation software ADAMS (automatic dynamic analysis of mechanical systems) and analyzed its dynamic behaviors. The results were consistent with those obtained from real-world experiments. Arnold et al. [20] built simulated disaster scenarios based on post-disaster satellite images and the actual locations of destroyed buildings to identify the most effective collaboration strategy for multi-robot SaR operations.

The other stream of work focuses on the training of robot operators. The data collected from the training process have been analyzed to understand human behaviors. Oviedo-Trespalacios [38] used a diver simulator to investigate participants’ reactions and behaviors under different traffic conditions. Bambakidis [39] presented a novel simulator for the training and rehearsal in microsurgery. Cherpillod [40] adopted a commercial flight simulator for immersive embodied interaction with a drone. Craighead [41] developed a simulated search and rescue environment, which can be used for the training of robot operators.

### 2.4. Knowledge Gaps

The literature review has revealed three knowledge gaps. First, despite the efforts made to develop robots to assist SaR operations, the existing robots are not adequate to perceive occluded spaces such as the voids buried in collapsed structures to pinpoint victims. UAVs and UGVs, equipped with vision-based sensors such as cameras and LiDAR, can only collect data from the surface of disaster areas, and are unable to see through unstructured occlusions. Snake-like robots are not efficient in searching a large area under time pressure because of their low moving speed and the complex and cluttered environment. Second, while many studies have been carried out in the searching of avalanche victims, the application of GPR, especially the hybrid use of GPR and UAV, has not been fully invesitgated in the more challenging scenario of urban SaR. Third, no simulation platform is yet available with various disaster scenarios to test new robots in acquiring both the above-rubble and below-rubble information, or to train users to work with the robots in disaster areas and interpret the robot-collected data for decision-making.

## 3. Robot Configuration

**Figure 1:**
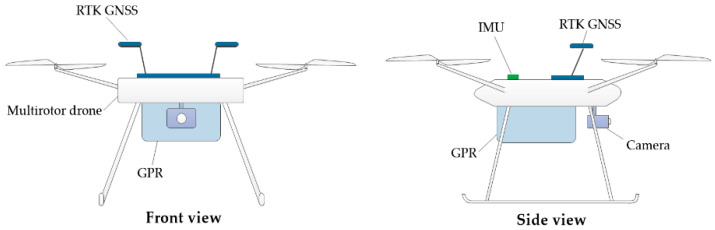
Conceptual diagram of the devised robot.

Figure 1 illustrates the configuration of a devised robot for urban SaR. Due to its high mobility and flexibility, a multi-rotor drone [42] is used as the platform to integrate different types of sensors. The on-board real-time kinematic (RTK) global navigation satellite system (GNSS) [43] can achieve a localization precision of centimeter-level, providing the robot with accurate spatial position information. The inertial measurement unit (IMU) [44] is used to measure the real-time accelerometer and angular velocity of the drone. The GNSS and IMU data are important for evaluating the robot state, and are indispensable for the robust and stable control of the drone. A high-resolution camera [45] is mounted to capture photos of surrounding environments, which is an essential information source to gain situational awareness of disaster areas. A GPR [46] is attached to the bottom of the drone, which emits electromagnetic waves to penetrate unstructured occlusions to sense the spaces under rubble. The GPR signal can penetrate through various media, including air, concrete, wood, water, etc. With different antennas, the penetrating depth ranges from 0.5 to 15 m. Due to the different conductivity and relative permittivity of different materials, the reflected signals received by the GPR antenna are sensitive to the structures under rubble. To be more specific, the strength of the reflected electromagnetic signal depends on the contrast of properties, such as dielectric constant *K*, of the different materials underground. In the case of urban SaR, the *K* of air is 1, while the *K* of construction materials such as concrete is usually around 6. This relatively large contrast of dielectric constant can cause strong reflections at the interface between the materials. The reflected signals, along with the two-way travel time, will then be received and calculated by a receiver of the antenna, based on which an A-scan of the current antenna location can be generated. As the antenna moves forward, multiple consecutive A-scans will be produced, which are then processed to form a two-dimensional (2D) image denoted by a B-scan. From this derived B-scan, the patterns of reflected signals can be recognized and interpreted to detect, locate, and characterize important features such as voids under the rubble that may contain survivors [47,48,49].

After a disaster, the robot can be teleoperated by a first responder to search the disaster area. The video captured by the onboard camera is streamed back to the operator’s computer screen in real time. Based on the provided visual information, the suspected rubble with a high probability of containing voids will be located by the first responder. Next, the first responder will teleoperate the robot to move along the rubble and use the GPR to scan the rubble. The below-rubble structures are reflected in the GPR scans, from which the first responder can determine whether a void exists.

**Figure 2:**
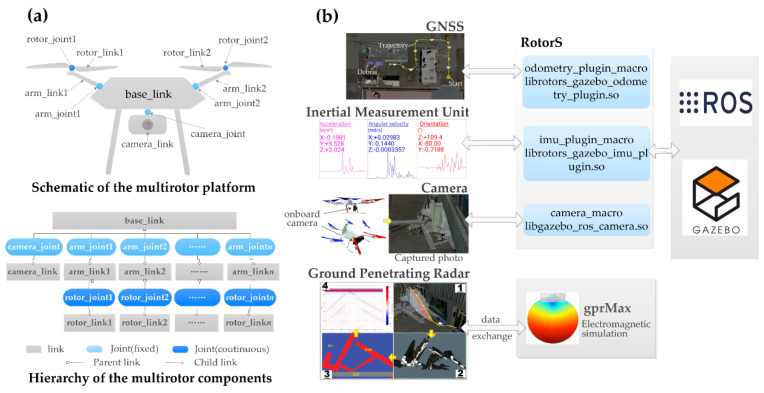
Simulation model for the proposed robot. (**a**) Structure of the multirotor platform, (**b**) simulation of the onboard sensors.

A simulation model of the robot is developed in a virtual environment. Gazebo is adopted as the simulation platform, and robot operating system (ROS) is used for robot software development. This work is based on RotorS, a bunch of ROS packages for UAV simulation that allow the developers to focus on higher-level tasks in a modular way, such as path planning and collision avoidance [50]. Figure 2 shows the schematic of the robot and how its basic components are simulated. The simulated multirotor platform is described by a unified robot description format (URDF) file. With this format, the structure of a robot is considered as a combination of two types of elements, i.e., links and joints. The former refers to the parts in a robot that are rigid bodies, while the latter denotes the connections between links, which define the relative movements of links. The process of developing a simulated multirotor platform is one that uses joints to assemble multiple links with different geometries and physical properties, as illustrated in Figure 2a.

Different sensor plugins, provided by the RotorS, are used to simulate the multiple onboard sensors. As shown in Figure 2b, the GNSS, the IMU and the camera are simulated, respectively, by the librotors_gazebo_odometry_plugin.so, the librotors_gazebo_imu_plugin.so, and the libgazebo_ros_camera.so. To attach the sensors to the drone platform, the corresponding macro files (i.e., odometry_plugin_macro, imu_plugin_macro, and camera_macro) should be referenced in the drone URDF file. The GPR is simulated by gprMax, an open-source software program for electromagnetic simulation. The GPR simulation procedure includes four steps. First, the coordinates of the flight path are recorded with special tags when the user activates the GPR to scan suspected rubble that has high probability of containing voids. Second, a cross-section of the scanned rubble along the flight path is obtained, given the tagged coordinates along the path. This is realized by a package in Unity3D, which allows users to obtain cross-section images of specific elements according to designated coordinate trajectories. With the obtained cross-section images, different colors are then manually assigned to the different elements in the images according to their materials. In our case, “red” is used for concrete slabs, “blue” is for the air, and “gray” is used for the ground. Material properties, such as dielectric constant and conductivity, also need to be specified. Next, the cross-section images are fed to gprMax, where the input is automatically processed to create geometric and mathematical models of the ruble based on the assigned colors and material properties. Finally, using the created models, synthetic GPR scans can be generated and communicated to the users for interpretation. Regarding the GPR simulation setup, the following GPR parameters are adopted. A frequency of 800 MHz was selected for the GPR antenna. Based on an analysis of the major components of urban disaster rubble, three types of materials were used in the simulation of rubble, i.e., air, concrete, and soil, which represent the void space in the rubble, the collapses columns and slabs, and the ground, respectively. The dielectric constant for the three materials is 1, 5.31 and 6 respectively, while the conductivity is 0, 0.03 and 0.01, respectively.

## 4. Robot Simulation Framework

A robot simulation framework is developed, which serves as a virtual testbed for the aerial robot devised in the last section and a training platform for users to learn to work with the robot in urban SaR operations. The developed platform should meet the following requirements. First, the simulated disaster scenarios should resemble real-world disaster scenes to provide users with a vivid and immersive experience. Second, the simulator should have an easy-to-use interface for users to manipulate the drone in the virtual disaster areas. Third, all the data collected by the simulated sensors should be saved with eligible formats, so that the data from different modalities can be integrated for interpretation.

### 4.1. Disaster Scenario Development

**Figure 3:**
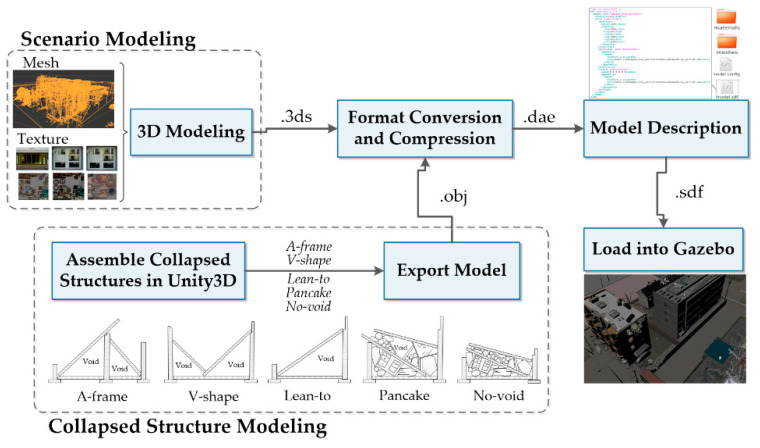
Virtual disaster scenario development workflow.

The disaster scenarios are developed in a virtual environment following two steps, i.e., the scenario modeling and the collapsed structure modeling. Figure 3 illustrates the overall workflow. The scenario is modeled based on photographs and videos of built environments destructed by disasters. 3D design software, e.g., 3D MAX, is used to build models that reflect the real post-disaster scenes. 3D meshes of the models are created and edited to represent the shapes and appearances of objects, such as damaged buildings and streets. Textures are attached to the meshes to improve fidelity. Since the simulation platform, Gazebo, only supports specific model formats with limited size, e.g. digital asset exchange (DAE) format, the created scenario models are then imported to Blender, where they are compressed and converted into another format that meets the requirements. In the final step, the paths of the meshes and textures, and other physical parameters of the models such as mass, inertial, and collision, should be defined in simulation description format (SDF) files. With the SDF files, the models can finally be loaded into Gazebo.

As for collapsed structure modeling, search and rescue manuals [51] are examined to identify typical collapsed structures with interior voids. Four types of collapse voids are included in the models: lean-to, A-frame, V-shape, and pancake [51]. In addition, collapsed structures with no void inside (denoted by no-void collapse) have also been modeled. The collapsed structures are modeled by assembling the rubble and debris prefabs provided in Unity3D. Then, they are exported from the Unity3D into a Wavefront object (OBJ) format. Similar to the process of scenario modeling, the exported OBJ models are also compressed by trimming unnecessary polygons and faces and converted into a DAE format. Finally, SDF files are created to describe the models so that Gazebo can recognize and load the models.

### 4.2. Robot Control

In this section, an easy-to-use controller for robot operation is developed, with which the users can manipulate the robot to perform SaR operations in virtual environments as they do in the real world.

**Figure 4:**
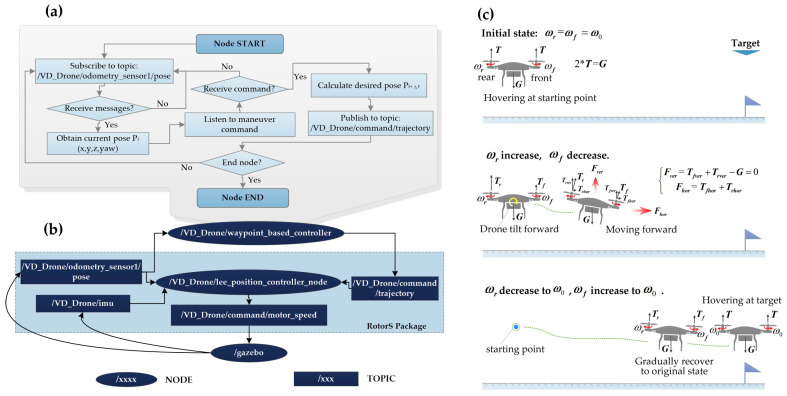
(**a**) Algorithm flowchart for node “/VD_Drone/waypoint_based_controller”, (**b**) a robot operating system (ROS) node graph that shows how our customized controller works, (**c**) schematic diagram of aerodynamics principle for drone maneuver—take moving forward as an example.

As shown in Figure 4a,b, the robot control is realized by combining a series of nodes and topics. In ROS, a node is an executable file that performs specific computation in the runtime, while a topic is a container of messages subscribed or published by a node. We developed a node, i.e., “/VD_Drone/waypoint_based_controller”, to directly handle the user input and forward the command messages to the nodes in lower layers. Once initialized, the node starts subscribing to the “/VD_Drone/odometry_sensor1/pose” topic, which conveys the sensory data that describe the current pose of the drone. Let (*x*,*y*,*z*) and *yaw* denote, respectively, the position coordinates and the orientation of the drone, and then its pose at time *t* can be described as $P_{t}(x,y,z,yaw)$. After a message is received from the topic, the node listens to the input event from a keyboard or a joystick to determine if any maneuver command is required. Four pairs of maneuvers are allowed, which are “go forward” or “go backward”, “go left” or “go right”, “go up” or “go down”, and “turn left” or “turn right”. Based on users’ commands, the desired pose at the next time step t+Δt is calculated. For example, if a “go forward” command is requested, the desired pose will be $P_{t+\Delta t}(x+\Delta x,y,z,yaw)$, where Δx is a preset constant value. Next, the desired pose is published to the “/VD_Drone/command/trajectory” topic. The loop keeps going until the user ends the program.

After the desired pose is published to the “/VD_Drone/command/trajectory” topic, a lower-layer node called “/VD_Drone/lee_position_controller_node” will take over the task. The node takes the pose (position, orientation) and IMU (accelerometer, and angular velocity) data as input to evaluate the current state of the drone. Based on the current state and desired pose, the rotating velocity of each rotor is calculated according to the aerodynamics. Take moving forward as an example (as shown in Figure 4c). At first, the drone is hovering at the starting point. This hovering state is maintained by rotating both the front and rear rotors with a speed $\omega_{0}$ that is just enough to produce thrusts (*T*) that can counteract the gravity (*G*). Let $\omega_{f}$ and $\omega_{r}$ denote the angular speed of the front and rear rotors, respectively. When a forward maneuver is required, the spinning speeds of the rear propellers, i.e., $\omega_{r}$, increase, while the ones of their front counterparts, i.e., $\omega_{f}$, decrease. The change of rotor speeds causes the rear thrust ($T_{r}$) to increase and the front thrust ($T_{f}$) to decrease, thus generating torque to tilt the drone forward. While the vertical components of the thrusts (i.e., $T_{rver}$ and $T_{fver}$) can still counteract the gravity (*G*), the horizontal components (i.e., $T_{rhor}$ and $T_{fhor}$) produce a net horizontal force (i.e., $F_{hor}$) to drive the drone moving forward. When the desired position is close, the drone orientation is gradually recovered to the original state by decreasing $\omega_{r}$ and increasing the $\omega_{f}$. During this process, the forwarding drone slows down gradually as it approaches the desired position. In the final step, the dynamically changing rotor speed is subscribed by the node “/gazebo”, which simulates and visualizes the movements of the drone in the virtual disaster scenarios.

### 4.3. Integration of Multimodal Simulation Data

**Figure 5:**
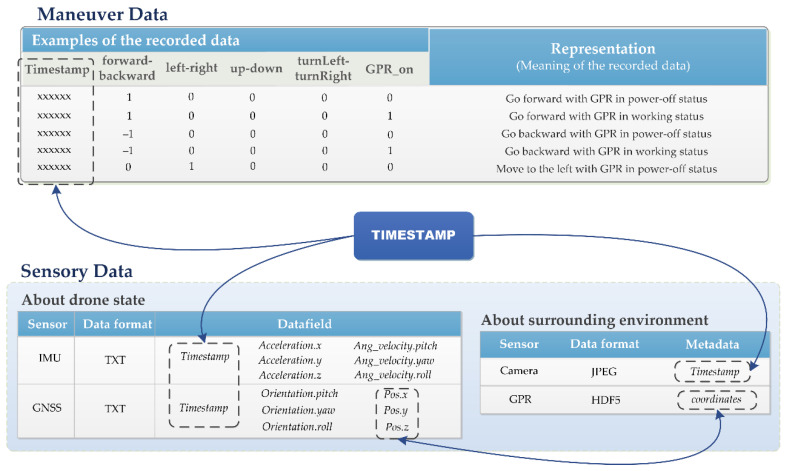
Schematic diagram for multimodal data fusion.

The data collected during the simulation process can be divided into three categories, i.e., data regarding maneuver commands, data regarding robot states (GNSS coordinates and IMU data), and data regarding environments (photos and GPR scans). Figure 5 illustrates the integration of these multimodal data. The maneuver data are recorded in a .txt file by five data fields, i.e., “forward–backward”, “left–right”, “up–down”, “turnLeft–turnRight”, and “GPR_on”. For the former four fields, number “1” represents the maneuver of “go forward”, “go left”, “go up”, and “turn left”, while “−1” indicates the opposites, and “0” indicates no action has been taken. For the last field (i.e., “GPR_on”), “1” is used to represent that the GPR sensor is activated to scan rubble, while “0” represents the GPR sensor is in a power-off status. Each piece of the data is tagged with a “*Timestamp*” to indicate the time when the maneuver is requested.

In terms of sensory data, GNSS and IMU collect data regarding the states of the drone, which are recorded separately in two different .txt files. The IMU data comprise linear accelerations (*Acceleration.x*, *Acceleration.y*, and *Acceleration.z*) and angular velocities (*Ang_velocity.pitch*, *Ang_velocity.yaw*, and *Ang_velocity.roll*), and GNSS data include position coordinates (*Pos.x*, *Pos.y*, and *Pos.z*) and orientation (*Orientation.pitch*, *Orientation.yaw*, and *Orientation.roll*). Both the IMU and GNSS data are tagged with *Timestamp*. The camera captures photos of the environments in JPEG format, and one field of the metadata (i.e., *Timestamp*) indicates when the photos are taken. The output of the GPR simulation is in HDF5 (hierarchical data format, version 5) format. The coordinates of the trajectory points, when GPR is used to scan a collapsed structure, are the metadata of the GPR simulation results. The recorded maneuver commands, and data collected by the IMU, the GNSS, and the camera are all time series data. As a result, data from these different modalities can be associated with each other using their timestamps. The GPR simulation results are directly linked with the GNSS data through coordinates, and can further be linked to data in other modalities via the timestamps.

## 5. Experimentation and Evaluation

### 5.1. Experiment Setup

Experiments were conducted to: (1) validate the efficacy of the GPR-integrated aerial robot in urban search and rescue, (2) evaluate the performance of using the simulation platform to train novice operators, and (3) analyze the recorded simulation data to extract useful insights about robot-assisted SaR operations. A workstation, DELL Precision Tower 7810, was used as the hardware platform, which is equipped with two Intel Xeon E5-2620 processors, a NVIDIA Quadro M2000 GPU, and 128 GB RAM. The distribution of ROS is Kinetic, and the version of Gazebo is 7.14.0. Both ROS and Gazebo were run on a virtual machine with the Ubuntu 16.04 system. A Sony DualShock 4 joystick was used as the robot control hardware.

In total, eight participants were invited to the experiments. To ensure the objectiveness of the experiments, all the invited participants are students without prior experience in search and rescue operations. They were neither familiar with the maneuver of drones nor the interpretation of GPR data. These similarities in participants’ background can mitigate the influences of different professional experience and skills on the performance, thus leading to a more objective evaluation. The eight participants were divided into two groups (each with four participants), i.e., the training group and the novice group.

Training group: The members of this group received training three times in a training scenario (scene #1 in Table 1) before a formal test was taken (scene #2 in Table 1).

Novice group: The members of this group took the formal test in scene #2 without any prior training.

Two different post-disaster scenarios have been developed respectively for the training purpose and formal test, as shown in Table 1. Scene #1 was designated for training. The types and locations of the collapsed structures in the scene were deliberately changed in different training sessions, while the total numbers of collapsed structures remained the same. This setting allows for objective assessment of the progress made by the participants as the number of training sessions increases. Both the training group and the novice group were required to take the formal test in scene #2, which contains six collapsed structures in total. The layout and the appearance of the collapsed structures in the scene were the same for both groups, so that the performances can be directly compared.

The three training sessions and the formal test follow the same procedures as elaborated in the following four steps.

Step 1. The participants were walked through the basic operation skills of the robot and the task they were going to undertake.

**Figure 6:**
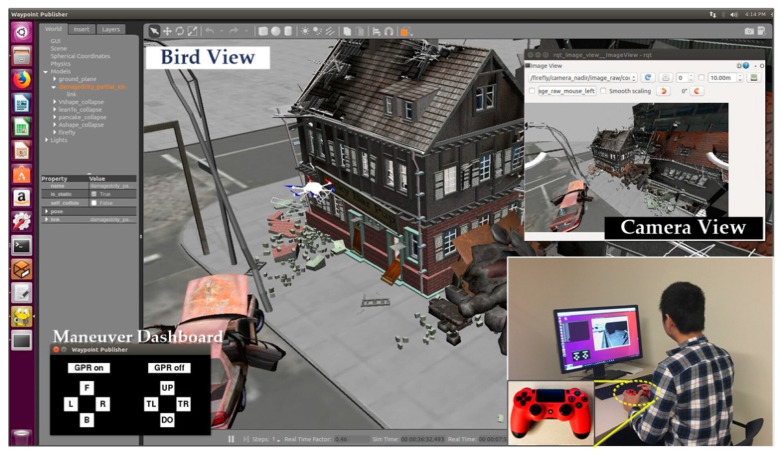
Experiment setup: Participants were required to operate the robot with a joystick to search for collapse voids based on the camera view.

Step 2. The participants carried out the first stage of the task, which required them to operate the robot with a joystick to search for collapsed structures in a simulated post-disaster scenario. In order to imitate the actual way of operating a real drone, the video stream, captured by the onboard camera (camera view in Figure 6), was the only source from which the participants can gain information about the disaster scenario. Once a suspected collapsed structure is located, the operator needs to trigger the “GPR on” button and maneuver the robot to fly across the rubble to generate a scan path for GPR simulation. When the participant believes all the suspected collapse structures have been found, the first stage of the task is finished.

Step 3. The time duration of Step 2 was recorded as the searching time. GPR simulation was then performed based on the scan paths.

Step 4. The second stage of the task was carried out, where the participants were provided with the photos of the collapsed structures captured by the onboard camera and the GPR simulation results. Based on the information, the participants were required to give their judgements on whether there would be voids under the rubble.

The participants’ performance was evaluated in the aspects of efficiency and effectiveness. The efficiency was measured by searching time, which refers to the time spent on searching the entire scene to detect suspected collapse structures. The effectiveness was measured by void-detection accuracy, which is the fraction of correct predictions given by the experiment participants on whether there is a void under rubble or not. Based on the evaluation metrics, the following hypotheses were formulated.

**Hypothesis** **1.** *The participants’ performance will improve as the number of training sessions increases, and the trained participants will significantly outperform novice participants who have not received training before.*

**Hypothesis** **2.** *After certain times of training, the trained participants will be able to work efficiently with the robot to locate voids.*

### 5.2. Experiment Results

(1) Validation of Hypothesis 1

**Figure 7:**
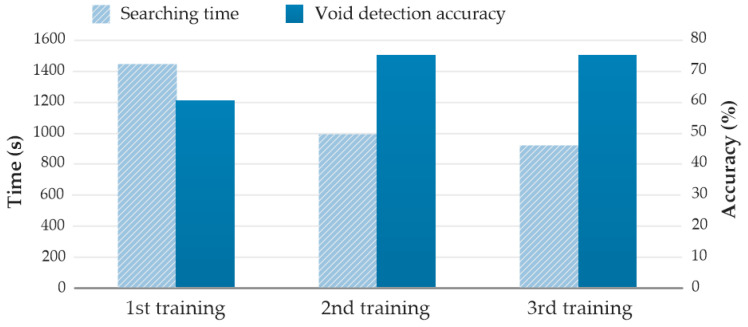
Performance improvement with the increase of training times.

Figure 7 presents the performance of the training group in different training sessions. The average searching time and void-detection accuracy of the four members in the first training session were 1449 s and 60%, respectively. The two metrics improved to 926 s and 75% during the third training session. The results validate Hypothesis 1, and indicate that receiving training in the simulation platform can help improve the users’ performance. It was observed that the performance improvement between the first training and the second training was more significant than that between the second and the third. The observation implies that the required knowledge and skills for operating the robot might have been mostly acquired through the first training.

**Figure 8:**
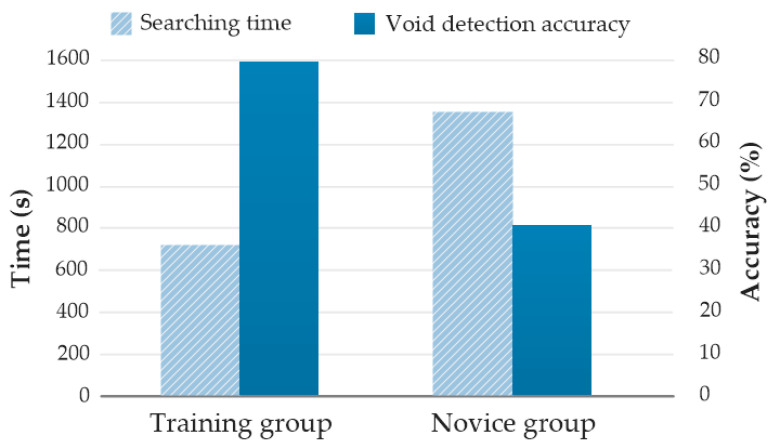
Performance comparison between the training group and the novice group in the formal test.

Figure 8 shows the performance of the training group and the novice group in the formal test. The average searching time and void-detection accuracy for the novice group were 1339 s and 41.7% respectively, while the training group attained on average 717 s and 79.2% for the two metrics. After three training sessions in scene #1, the training group outperformed the novice group by saving 622 s in searching time, and improving 37.5% in void-detection accuracy.

(2) Validation of Hypothesis 2

To evaluate the efficacy of the devised robot in assisting urban SaR, we analyzed the results of the training group in the formal test. On average, it took 717 s for the group to complete the search in scene #2 (3120 m^2^), that is, roughly 165 h per square mile when only one robot is used. According to Reference [52], it normally takes 924 man hours to search one square mile using a grid search. This result demonstrates the great potential of using the robot to save time in searching a large disaster area. After training, the average void-detection accuracy was 79.2%. To the best of the authors’ knowledge, no prior research has sought to quantitatively evaluate the effectiveness of a robot in assisting first responders to detect collapse voids; hence, the average void-detection accuracy obtained in our study can serve as a benchmark against which future studies can measure their outcomes.

**Figure 9:**
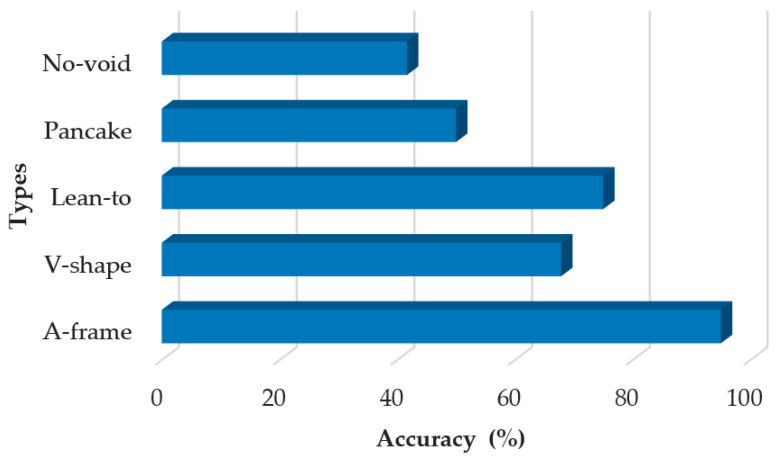
Void-detection accuracy distribution of different collapse types.

Figure 9 presents the void-detection accuracy for different types of collapse structures listed in Table 1 (including all the collapse structures used in the three training sessions and the formal test). It is observed that the void spaces under A-frame collapse structures have been identified with the highest void-detection accuracy (95%). Ranked in the second and the third place are the lean-to (75%) and the V-shape (67.9%), respectively. The void-detection accuracy for the pancake collapse and the no-void collapse was, respectively, 50% and 41.7%, which is not satisfactory.

**Figure 10:**
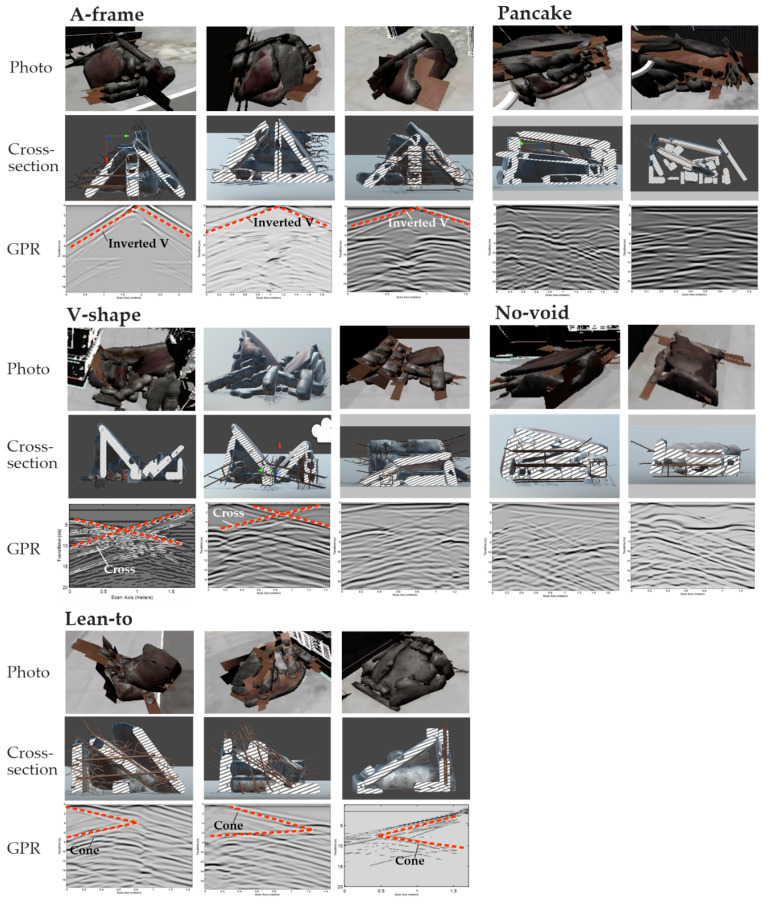
Examples of photos, cross-sections, and GPR simulation results of different types of collapses.

To investigate causes of this variance of the detection accuracy, the photos, cross-section images, and GPR simulation results of the different types of collapses were extracted for analysis. As shown in Figure 10, the A-frame collapse, V-shape collapse, and the lean-to collapse show consistently distinct patterns in both photos and GPR scans, which are an inverted V-shape, a cross-shape, and a cone-shape, respectively. With these distinctive patterns, it would be relatively straightforward for users to correlate the patterns with the void spaces they represent. Take A-frame collapse as an example: The inverted V highlighted by red dotted lines in the GPR profiles in Figure 10 corresponds to the two leaning concrete slabs, while the horizontal straight lines below indicate the location of ground. One should notice that the horizontal lines are interrupted in the middle, which is caused by the supporting columns in the middle. On the contrary, it is relatively difficult to tell the differences between the pancake collapse and the no-void collapse from the photos and GPR scans. As a result, the participants might mistake a pancake collapse for a no-void collapse, and vice versa. In future applications, drone operators can be allowed to scan the same collapse from different directions to obtain multiple GPR B-scans. These B-scans provide subsurface information of the collapse from different perspectives, and thus might be helpful to improve first responders’ performance in void recognition.

### 5.3. Analysis of Recorded Simulation Data

In this section, the recorded multimodal data are extracted for further analysis to get insights into the process of robot-assisted SaR operations, and understand how the training process helped improve the participants’ performance.

**Figure 11:**
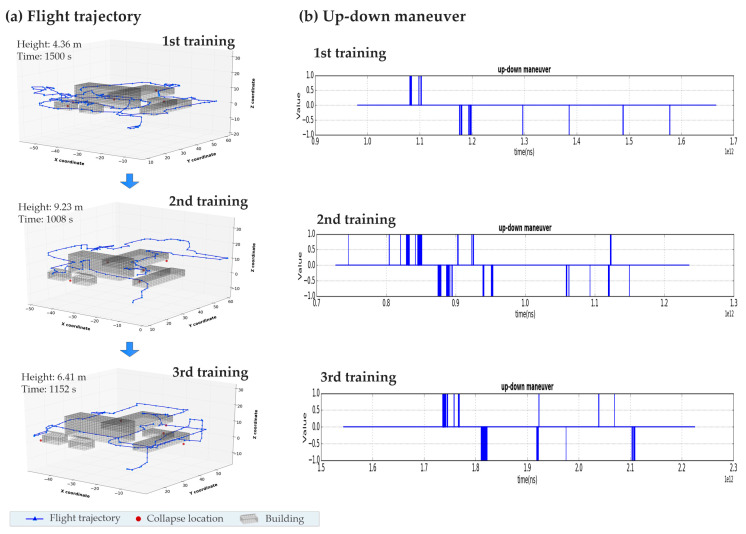
Performance evolvement of a participant during the three training sessions. (**a**) Flight trajectories of the robot’s movements, (**b**) up-down maneuver requested by the participant.

Figure 11 presents the performance evolvement of a participant during the three training sessions. In the first training session, the trajectory of the participant was choppy and stayed at a low-altitude (LA) area. The average height of the flight trajectory was 4.36 m while the total searching time was 1500 s. The time performance was significantly improved in the second training, in which the participant used 1008 s to complete the searching. The flight trajectory became much smoother and the average height increased to 9.23 m. No further improvement in time performance was observed in the third training. This evolvement process indicates that the participant, in the second training, had acquired an explicit searching strategy that first looks over the whole scenario at the high-altitude (HA) area and then closely observes and scans the rubble at the low altitude. We denote the searching strategy by a HA strategy. The adoption of the HA strategy is also reflected by the frequency of the up–down maneuver (Figure 11b). In the first training session, the command of going up (down) was rarely requested, while the maneuver was frequently taken in the second and third training to navigate the drone between the HA area and the LA area.

**Figure 12:**
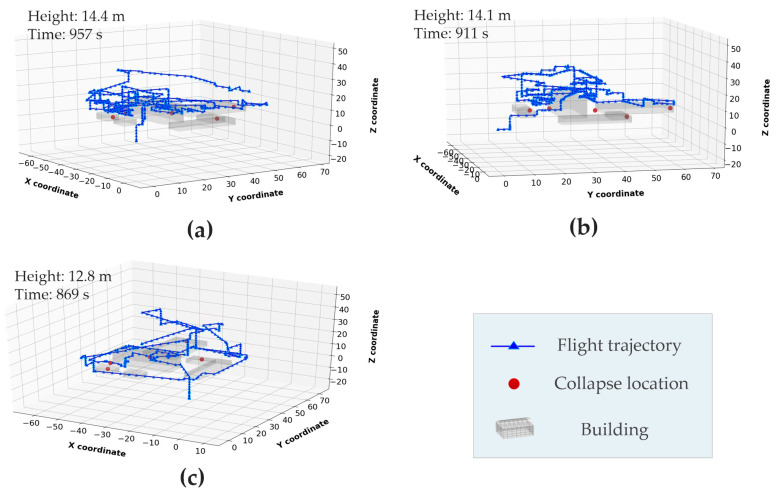
Searching path of three novice participants applying the high-altitude (HA) strategy. (**a**) Novice participant 1, (**b**) novice participant 2, (**c**) novice participant 3.

The adoption of the HA searching strategy might be an important factor that contributes to the efficiency improvement. By escalating to a high altitude, operators can grasp the scenario in a holistic way and locate suspected collapsed structures in a shorter time. With this strategy, the operators can also save time by avoiding the detours between the buildings. To validate this assumption, an additional experiment was implemented, where three novice participants were recruited to repeat the first training session in Table 1. The newly recruited participants were told in advance to apply the HA strategy; other than this, they have the same background as those recruited in Section 5.1. Figure 12 shows the flight trajectories of the three new participants. The average height of the searching path was 14.4 m, 14.1 m and 12.8 m, respectively, for the three participants, which implies that the HA strategy has been strictly followed. The average time spent on searching the whole scene was 912 s, which was significantly lower than the average searching time (1449 s) of the first training session in Figure 7. This demonstrates the enormous contribution of the HA searching strategy to improving the efficiency and expediting the SaR operations. However, it should be noted that the time performance (912 s) we obtained in the additional experiment was even slightly better than that of the third training (926 s) in Figure 7. This observation indicates that there are other factors (e.g., familiarity with scene layout and acquaintance of operation skills) that have also contributed to the efficiency improvement; nevertheless, their contributions are not as significant as the grasp of the HA searching strategy. Since all the participants did not necessarily grasp the HA strategy during training, their performance improvement was partially driven by those less significant factors (e.g., familiarity with the scenario and operation skills). Therefore, it is no wonder that the original participants, despite the three training sessions they went through, only ended up with a time performance close to the novice participants in our additional experiment, who were taught to follow the HA strategy from the beginning.

### 5.4. Discussion

The efficacy of the robot was validated by the experiments. After three training sessions, the participants attained an average void-detection accuracy of 79.2% with a searching efficiency of 165 h per square mile. The results demonstrate that the first responders can collaboratively work with the robot to gain situational awareness of both the above-rubble and below-rubble spaces, and thus locate buried voids in a timely manner. A variance of the void-detection accuracy among the different types of collapsed structures was observed, which indicates that the pancake collapse is more likely to be mistaken for a no-void collapse compared to other collapse types. The observation underscores the need of special focus on pancake collapse in SaR operation; otherwise, the void spaces under the pancake rubble could be missed, leaving the entrapped victims in danger.

The developed simulation framework can act as a platform for training first responders in working with SaR robots. After training in the simulation platform, the participants’ performances were significantly improved by saving 523 s in searching time and an increase of 15% in void-detection accuracy. In the formal test, the trained participants correctly identified 79.2% of the voids in 717 s, which outperformed the novice participants (1339 s and 41.7%). Based on the fact that the ranges of improvements were much more significant between the first and the second training sessions than that between the second and the third, it is deduced that most of the required knowledge and skills for operating the robot would have been acquired through the first training session.

The recorded data of the training process provided useful insights into the SaR operation. By analysis, we found that the performance improvement during the training was partially attributed to the acquisition of a new searching strategy that first looks over the whole scenario at high altitude (HA) and then closely observes and scans the rubble at low altitude. It was observed that the high-altitude (HA) searching strategy cost less time than the constantly low-altitude (LA) searching. The observation implies that the HA strategy tends to yield a more efficient SaR operation than the LA strategy.

The application of the developed simulator can be extended to the following two aspects in the future. First, the simulator can be used to compare and evaluate the efficiency of different searching strategies, thus providing essential information to enable efficient SaR operations. This has been preliminarily demonstrated by the comparison of the HA and LA searching strategies, which implies a higher efficiency of the HA strategy. In the future, many other searching strategies can be tested in the simulation platform to answer a series of questions that are normally difficult to address in real life. For example, when multiple drones are to be deployed in an urban disaster site, how many drones are enough and what strategies should the drones follow to cooperate with each other to yield efficient SaR operations? Second, the simulation platform has great potential to be used for training autonomous robots. A massive amount of paired data (maneuver commands versus environment information) can be generated by inviting more participants. From the maneuver–environment paired data, SaR robots can learn how a human operator makes decisions (different maneuvers such as moving forward, turn left, and turn on GPR) given a dynamically changing environment, and optimize the strategy for searching a new environment. In addition, reinforcement learning can also be adopted, which trains a robot by allowing it to explore a virtual scenario under a preset reward mechanism.

Further research efforts are needed to address the following limitations.

First, the onboard ground-penetrating radar is not directly simulated in Gazebo, but in an external software program—gprMax. Thus, the scan path and the corresponding cross-section of the rubble need to be imported from Gazebo to gprMax. This cross-platform data interaction is currently realized by manual operations. This inefficient process needs to be further automated by developing a data exchange interface.

Second, the GNSS of the devised robot is currently simulated by a ground-truth odometry sensor provided by Gazebo. This is not the case in reality, where the GNSS positioning is affected by many factors (e.g., signal occlusion) and always has a certain level of deviation from the ground truth.

Third, computer algorithms should be further developed and incorporated to assist first responders in void recognition. Given the complexity of the subsurface condition and the resulting GPR profiles, it would be difficult to solely depend on human observers to interpret the raw GPR scans and photos of rubble for void recognition. Our previous work [47] developed an automated algorithm to detect voids from GPR data. In the future, algorithms of this type and other techniques such as computer vision [53] should be incorporated as part of the system to support first responders’ decision-making. In addition, for simplicity, this research only considers mere voids without entrapped humans. Future research should focus on analyzing and evaluating the effects of the entrapped humans on the corresponding GRP profiles.

## 6. Conclusions

Timely and effective search and rescue (SaR) operations in urban environments are crucial for mitigating fatalities brought by natural or manmade disasters. Despite the advancements in robotics, existing SaR robots fall short of sensing the occluded spaces under the rubble in a rapid and reliable manner. To gain rapid and comprehensive situational awareness, this study proposed a framework to simulate an unmanned aerial vehicle (UAV) with multiple sensors to acquire both above-rubble and below-rubble information for post-disaster SaR in virtual environments. A robot was devised to detect voids under the rubble by integrating a UAV platform with a ground-penetrating radar and conventional sensors (e.g., GNSS, IMU, camera). A simulation platform was developed for testing the SaR robot and training novice operators in a virtual environment. Experiments were conducted for validation purposes. The results demonstrate that the devised robot can assist rescue personnel to locate below-rubble void spaces with an accuracy of 79.2% in a timely manner. The capability of the simulation platform in novice training has also been demonstrated via the significant improvement attained by the participants.

Useful insights have been extracted from the datasets resulting from the experiments. First, voids under pancake collapsed structures are more difficult to identify compared with other typical collapse types (A-frame, V-shape, and lean-to); hence, special attentions should be paid to this collapse type in SaR operations to determine the existence of voids. Second, the high-altitude (HA) strategy could yield a more efficient outcome than the constant low-altitude (LA) strategy when an aerial robot is applied in SaR operations.

## Figures and Tables

**Table 1:** The developed post-disaster scenarios for the experiments.

		Scene #1			Scene #2
Picture		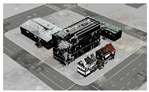			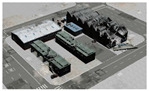
Purpose		Training			Formal test
		1st	2nd	3rd	
Number of collapses	A-frame	1	1	1	1
	V-shape	1	1	1	2
	Pancake	1	1	0	1
	Lean-to	0	1	2	1
	No-void	2	1	1	1
